# Supplementary material for: Conditional chemoconnectomics (cCCTomics) as a strategy for efficient and conditional targeting of chemical transmission
Source: eLife. 2024 Apr 30;12:RP91927. doi: 10.7554/eLife.91927 (PMC11060718; doi:10.7554/eLife.91927)
Supplement: Figure 3—source data 3. [file elife-91927-fig3-data3.zip › Figure 3 source data3 part 1.zip!/Figure 3-Source data 3-part1/1067-9-CRISPResso_Report_c33kf3u3.zip!/CRISPResso_on_1067_10/3b.Insertion_deletion_substitutions_size_hist.pdf]

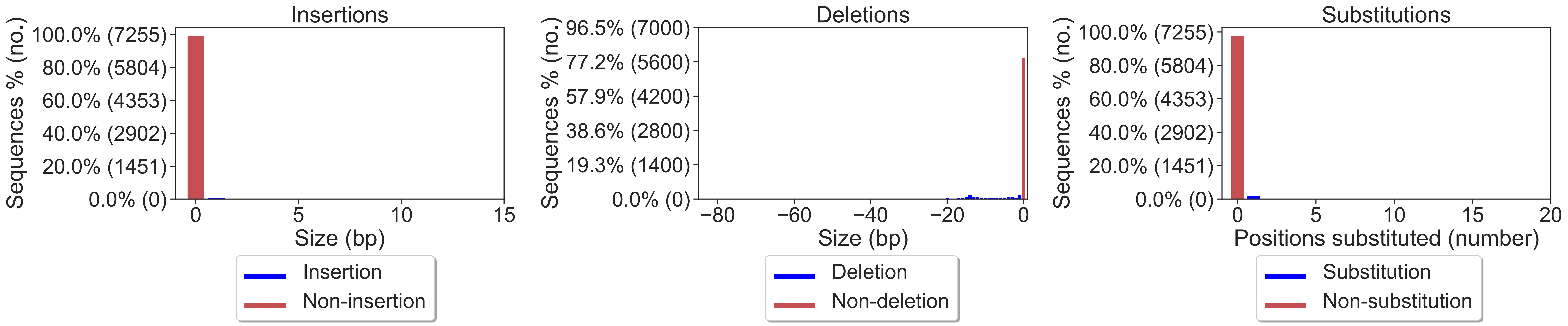

Insertions
Sequences % (no.)
100.0% (7255)
80.0% (5804)
60.0% (4353)
40.0% (2902)
20.0% (1451)
0.0% (0)
0
5
10
15
Size (bp)
Insertion
Non-insertion
Deletions
Sequences % (no.)
96.5% (7000)
77.2% (5600)
57.9% (4200)
38.6% (2800)
19.3% (1400)
0.0% (0)
−80
−60
−40
−20
0
Size (bp)
Deletion
Non-deletion
Substitutions
Sequences % (no.)
100.0% (7255)
80.0% (5804)
60.0% (4353)
40.0% (2902)
20.0% (1451)
0.0% (0)
0
5
10
15
20
Positions substituted (number)
Substitution
Non-substitution
